# Experiences and Perceptions of Medical Cannabis among People Living with Chronic Pain and Community Pharmacists: A Qualitative Study in Canada

**DOI:** 10.1080/24740527.2023.2258537

**Published:** 2023-09-13

**Authors:** Lise Dassieu, Emilie Paul-Savoie, Élise Develay, Ana Cecilia Villela Guilhon, Line Guénette, Kadija Perreault, Hélène Beaudry, Laurent Dupuis, Claudie Audet, Anaïs Lacasse

**Affiliations:** a Research Center of the Centre hospitalier de l’Université de Montréal, Montreal, Quebec, Canada; b Department of Biomedical Sciences, Faculty of Medicine, Université de Montréal, Montreal, Quebec, Canada; c Quebec Pain Research Network, Sherbrooke, Quebec, Canada; d School of Nursing, Faculty of Medicine and Health Sciences, Université de Sherbrooke, Longueuil, Quebec, Canada; e Department of Community Health Sciences, Faculty of Medicine and Health Sciences, Université de Sherbrooke, Longueuil, Quebec, Canada; f Faculty of Pharmacy, Université Laval, Quebec City, Quebec, Canada; g Centre de recherche du CHU de Québec, Université Laval, Axe Santé des populations et pratiques optimales en santé, Quebec City, Quebec, Canada; h Centre interdisciplinaire de recherche en réadaptation et en intégration sociale (CIRRIS), Centre intégré universitaire de santé et de services sociaux de la Capitale-Nationale, Quebec City, Quebec, Canada; i Department of Rehabilitation, Faculty of Medicine, Université Laval, Quebec City, Quebec, Canada; j Department of Health Sciences, Université du Québec en Abitibi-Témiscamingue, Rouyn-Noranda, Quebec, Canada

**Keywords:** cannabis, chronic pain, experience, perspectives, pharmacists, qualitative research

## Abstract

**Background:**

The use of cannabis to treat chronic pain is under debate despite high expectations from patients. Qualitative data obtained by exploring both patients’ and health professionals’ perspectives are scarce.

**Aims:**

This study aimed to understand the experiences and perceptions of people living with chronic pain and community pharmacists regarding the role of cannabis in chronic pain treatment in the Canadian context where both medical and recreational cannabis are legal.

**Methods:**

We conducted 12 online focus groups (July 2020–February 2021) with 26 patients and 19 community pharmacists using semistructured discussion guides. All discussions were audio recorded and transcribed verbatim were analyzed using a reflexive thematic approach.

**Results:**

We developed three themes related to patients’ perspectives and three themes related to pharmacists’ perspectives. Patients’ perspectives included (1) cannabis as an alternative to other pain medications, (2) a new treatment with potential health-related risks, and (3) a therapy rather than a recreational drug. Pharmacists’ perspectives included (1) challenges in monitoring drug interactions with cannabis in the context of scarce research data, (2) informing and treating patients self-medicating with cannabis amid its growing popularity, and (3) financial costs and legal constraints for patients.

**Conclusions:**

This study highlights patients’ and pharmacists’ urgent need for reliable information regarding the benefits and risks of cannabis. Training tailored to pharmacists’ needs and evidence-based information for patients should be developed to support pharmacists’ practice, improve patients’ experiences, and promote safe cannabis use.

## RÉSUMÉ

**Contexte:** L'utilisation du cannabis pour traiter la douleur chronique fait l'objet d'un débat, malgré les fortes attentes des patients. Les données qualitatives issues de l’exploration des perspectives à la fois des patients et des professionnels de la santé, demeurent rares.

**Objectifs:** Cette étude visait à comprendre les expériences et les perceptions des personnes vivant avec une douleur chronique et des pharmaciens communautaires concernant le rôle du cannabis dans le traitement de la douleur chronique dans le contexte canadien, où le cannabis médical et récréatif est légal.

**Méthodes:** Nous avons mené 12 groupes de discussion en ligne entre juillet 2020 et février 2021, réunissant 26 patients et 19 pharmaciens communautaires, à l'aide de guides de discussion semi-structurés. Toutes les discussions ont été enregistrées et transcrites mot à mot, puis analysées à l'aide d'une approche thématique réflexive.

**Résultats:** Nous avons développé trois thèmes liés aux perspectives des patients et trois thèmes liés aux perspectives des pharmaciens. Le point de vue des patients incluait (1) le cannabis comme option de rechange à d'autres médicaments contre la douleur, (2) un nouveau traitement avec des risques potentiels pour la santé, et (3) un traitement plutôt qu'une drogue récréative. Le point de vue des pharmaciens portait sur (1) les défis liés à la surveillance des interactions médicamenteuses avec le cannabis dans le contexte de la rareté des données de recherche, (2) l'information et le traitement des patients qui s'auto-médicamentent avec du cannabis dans un contexte de popularité croissante, et (3) les coûts financiers et les contraintes légales pour les patients.

**Conclusions:** Cette étude met en évidence le besoin urgent des patients et des pharmaciens de disposer d'informations fiables sur les avantages et les risques du cannabis. Une formation adaptée aux besoins des pharmaciens et des informations fondées sur des données probantes pour les patients devraient être développées pour soutenir la pratique des pharmaciens, améliorer l'expérience des patients et promouvoir une utilisation sûre du cannabis.

## Introduction

The use of medical cannabis in the treatment of chronic pain is currently controversial and under debate. In 2021, based on a set of literature reviews,^1–4^ the International Association for the Study of Pain pointed out the absence of a scientific consensus demonstrating the safety and efficacy of cannabis and cannabinoids in the treatment of chronic pain. Given this insufficient evidence and the previously documented adverse effects of heavy cannabis use,^5^ the International Association for the Study of Pain did not recommend the general use of cannabis products in pain management until the clinical and preclinical research gaps are filled.^6^ However, a recent systematic review reported small pain improvements.^7^

Despite the presence of published position statements and concerns, patients with chronic pain hold positive attitudes toward cannabis products.^8,9^ A review of patients’ perceptions of medical cannabis for chronic musculoskeletal pain showed that patients using medical cannabis were overall satisfied with this treatment and tended to describe more benefits than harms.^9^ According to a survey held in an Ohio hospital, a large majority of respondents living with chronic pain were considering the use of medical cannabis.^10^ In a recent Canadian study, 30% of persons living with chronic pain used cannabis to relieve their pain.^11^ Younger patients as well as those with higher pain intensity and interference were more likely to use cannabis for pain.^11,12^ Other research suggested the existence of diverse profiles of patients using cannabis for chronic pain, with different preferences and intensity of utilization.^13^

Recent literature suggests that although many physicians had an overall positive image of medical cannabis,^14,15^ only a small proportion of them were likely to recommend it to chronic pain patients.^14–17^ The practices of other health care providers involved in chronic pain management, such as pharmacists, remain poorly documented.^18^ In a study of patient decision making regarding cannabis use for chronic pain, only a small proportion of participants received advice from a health care provider regarding choice of cannabis products, whereas more than half relied on cannabis retailers’ advice.^13^ Because pharmacists are highly involved in the pharmacological treatment of chronic pain in primary care,^19–21^ it is essential to document their experiences and needs.

Qualitative insight into patients’ and pharmacists’ experiences with cannabis in chronic pain treatment would highly improve knowledge of their diverse perspectives, practices, and challenges. This study aimed to understand patients’ and pharmacists’ experiences, perceptions, and concerns regarding the use of cannabis for chronic pain. This article will bolster the identification of patients’ and pharmacists’ needs for support and information regarding cannabis and associated benefits and risks in chronic pain treatment.

## Methods

The methods are reported following the Standards for Reporting Qualitative Research guidelines for qualitative research.^22^

### Study Setting and Design

This study took place in the Canadian province of Quebec, where medical cannabis has been available in specialized facilities upon authorization from a physician since 2001 and recreational cannabis has been legal and sold in government-owned retail stores since October 2018. This article is part of a larger qualitative study featuring 12 online (Zoom)® focus groups aimed at understanding the experiences of people living with chronic pain (*n* = 26 participants, six focus groups) and community pharmacists (*n* = 19 participants, six focus groups) regarding the adverse effects of analgesic drugs. Cannabis use was deeply discussed by participants, therefore warranting a standalone analysis and report. In this study, the term “medical cannabis” encompasses raw herbal cannabis (plant), cannabis extract from the plant, and cannabinoids, which constitute a group of chemicals that activate cannabinoid receptors in the human body.^23^ For an extensive description of the methods and further findings, see our other publication.^19^

### Recruitment and Participants’ Characteristics

All participants provided written informed consent to participate in the study. The study was approved by the Research Ethics Boards of the Center hospitalier de l’Université de Montréal (No. 19.367) and the Université du Québec en AbitibiTémiscamingue (No. 202003, Lacasse, A). After the focus groups, all participants received a CA$75 honorarium in recognition for their time and effort.

#### People Living with Chronic Pain

Individuals living with chronic pain were recruited among the participants of a prospective cohort study aimed at examining the pharmacological, physical, and psychological treatment of chronic pain in Quebec (*n* = 1935).^24^ The eligibility criteria applied to that cohort for the present study were (1) living with pain for 6 months or more, (2) reporting using pain medications, and (3) reporting one or more moderate to severe drug adverse effects. Among cohort participants who agreed to be recontacted for other studies (*n* = 1114), a total of 150 eligible participants were sent an email inviting them to take part in an online focus group with other people living with chronic pain (purposive sampling). Those who agreed to participate were recontacted by phone and given an appointment. Among the 26 recruited participants, 12 were women and 14 were men; participants’ ages varied between 34 and 82 years, and most of them had lived with pain of moderate to severe intensity (≥4/10) for more than 10 years (24/26). Eleven out of 26 participants reported using or having used cannabis or cannabinoids to relieve their chronic pain. Nonusers also shared their perspectives on cannabis during the focus groups.

#### Pharmacists

All Quebec pharmacists working either in community pharmacies or in family medicine groups (i.e., multidisciplinary primary care facilities^25^) were eligible to participate in the focus groups. They were reached using various recruiting strategies including dissemination through provincial professional associations, social media, the research team’s network, and the snowball method. Of the 19 recruited pharmacists, 11 were women and 8 were men, and the age range was 26 to 56 years. Eight worked in community pharmacies only and 11 had a mixed practice in community pharmacies and family medicine groups; 14 had less than 10 years of practice.

### Data Collection

Data collection took place between July 2020 and February 2021. The focus groups were conducted by two experienced facilitators with professional backgrounds in sociology (E.D.) and psychology (A.C.V.G.). Discussions with participants lasted 90 to 120 min and were audio recorded. Recordings were then transcribed verbatim. Participants were given pseudonyms during the discussions to preserve their anonymity. The focus groups were conducted in French, the main language used in the province of Quebec. Only quotes selected for this article were translated to English by bilingual members of the research team.

The semistructured discussion guide for people living with chronic pain focused on their general experiences with the adverse effects of pain medication. The guide included specific prompts related to their experiences and perspectives regarding cannabis. The semistructured guide for pharmacists covered their practices and challenges in managing and preventing adverse effects of analgesics, with a question focusing on cannabis. Complete interview guides are available as supplementary materials included in our previous publication.^19^ Data collection and analysis began concurrently in a back-and-forth movement to explore some specific domains more deeply.^26^ In particular, cannabis was central to the initial discussions. Therefore, we further explored it in the focus groups through systematic questions and prompts.

### Data Analysis

We analyzed cannabis-related data using a reflexive thematic approach.^27^ The analysis was conducted in French by three members of the research team: it was spearheaded by L. Dassieu, a health sociologist with profound proficiency in qualitative research encompassing chronic pain, substance use, and social inequities, working in conjunction with E. Develay, who has a background in sociology, and A. C. Villela Guilhon, with a background rooted in psychology. The anticipation was that their collective expertise would facilitate comprehension of pivotal facets pertaining to the social phenomenon of cannabis consumption. During the analysis, a constructivist and inductive approach was employed to formulate the themes.^27^ Triple coding of the data fostered the researchers’ reflexivity and enriched the interpretations. The analysts used NVivo version 12 (Lumivero, Denver, CO, USA) to manage data and code excerpts into different labels developed iteratively.^27^ The lead analyst (L.D.) constructed the final cannabis-related themes by integrating and articulating the codes into a logical and consistent framework. Regular team meetings enabled discussion of our diverse insights into the data. Memo writing supported data interpretation throughout the analysis process and the development of the codes and themes.

## Results

**Figure 1.**
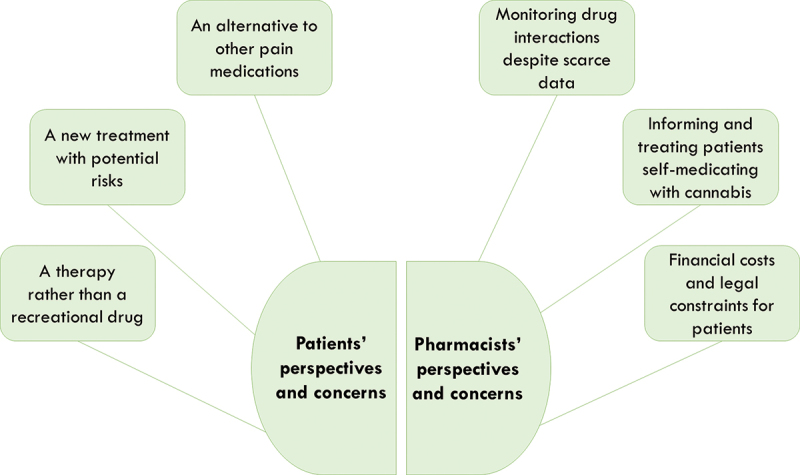
Cannabis use in chronic pain treatment: understanding patients’ and pharmacists’ perspectives.

The first section of the results focuses on patients’ experiences and perspectives on cannabis in chronic pain treatment; the second section is dedicated to pharmacists’ experiences and perspectives. Each section includes three themes reflecting the main issues raised by participants. A graphical representation of the results is shown in Figure 1.

### Patients’ Perspectives

During the focus groups, patients discussed various types of medical cannabis products, especially cannabidiol (CBD) oil and a prescribed synthetic cannabinoid medication (nabilone). Most patients using cannabis had a prescription or a physician authorization to access medical cannabis. A few participants tried cannabis products via the licensed provincial retail stores selling recreational cannabis, before asking for medical advice.

#### An Alternative to Unsatisfactory Pain Medications

Several patients reported that they decided to look for information about medical cannabis because they were unsatisfied with their pain medications. Indeed, several participants using cannabis products for their chronic pain found that the benefit–risk ratio was overall better than that for other pain medications. According to these participants, cannabis, especially CBD, was more effective in reducing their pain and had fewer adverse effects than other pharmacological treatments such as opioids or antidepressants:

The side effects of painkillers are so heavy that the side effects of cannabis, for me, are minimal, it’s not even on the same gradient of evaluation. I would have to use a huge amount of cannabis to have the same level of side effects that I have on the other side. (Male, 60–69, FG6)

Many patients found that cannabis use was less concerning than opioid use in terms of potential risks and harms. Indeed, several participants pictured cannabis as a “natural” or “plant-based” product, seen as less harmful than “chemical” or “synthetic” medications. One participant described other pain medications as dangerous narcotics, as opposed to cannabis:

The real [dangerous] drug^a^^a^Translation note: In French, the term *drogue* is used for illicit substances only and often has a negative connotation (e.g., danger, or illegality), which is the case in this excerpt. The English translation “drug,” which refers to both illicit substances and medications, does not render this important nuance. isn’t pot, it’s not medical cannabis, it’s the medication that the pharmacist gives us, that’s the real [dangerous] drug. (Female, 50–59, FG2)

Furthermore, some participants hoped that cannabis could be a suitable alternative to opioids, enabling them to taper off or cease these medications:

I should try cannabis again, CBD or BDC [*sic*], to see what effect it would have on me. Maybe it would help me more than [hydromorphone], I don’t know. (Male, 60–69, FG2)

Compared to tramadol, which I have incredible sleep problems with, where I’m stoned and I’m not there at all and I can’t drive my car. … No! I’m trying to completely cut out everything else and just go on with CBD. (Female, 50–59, FG2)

#### Uncertainties and Fears: A New Medication with Potential Risks

However, not all patients had positive views on cannabis. Some were hesitant to try it and expressed worries regarding a treatment they perceived as “new”:

Sometimes, I have the impression of being like a Guinea pig, you know, the canna … all that is related to cannabis, it was new for a while, the effects [were unknown], and I was afraid. In the beginning, before it became popular, there was a medication that I was the only one taking in the whole area here. (Female, 40–49, FG6)

Several patients expressed the need for more research to get a better idea of the potential of cannabis as a pain treatment. They hoped that scientists would put medical cannabis and cannabis-based medications on their agenda:

Is there any research being done? It would be nice to have some research done. I know that some chemists have started to work a little on CBD, but it doesn’t seem like there are any big developments in this area, compared to the pharmaceutical companies who are working on a lot of drugs. I think it’s an avenue that should be explored, but I don’t hear about a lot of development in that area. (Male, 60–69, FG3)

Other participants reported negative experiences with cannabis products. Nabilone, the synthetic cannabinoid delivered in pharmacies upon prescription, disappointed several participants due to inconvenient adverse effects such as intense fatigue:

I tried cannabis on my doctor’s advice. Not the cannabis that is sold in companies now or on the street, but, I mean, cannabis in the form of a medication that was supposed to be very good for pain. I tried that for several months, but it really prevented me from functioning normally, I was amorphous, very bad, physically and mentally. (Male, 80–89, FG6)

Several patients did not wish to try cannabis. Fear was their predominant feeling. For example, one of them explained that, in her opinion, cannabis remained a dangerous substance even when medically prescribed:

I’m afraid of it [cannabis]. No one ever told me that an opioid was a narcotic. I think if they had told me at the beginning, I would have refused to take it. I’ve never used [illicit] drugs, so for me, I was already afraid of it. The fact that, for me, cannabis is also on the [illicit/dangerous] drugs side makes me not interested in trying it at all. (Female, 40–49, FG6)

Some other patients were dissuaded from using cannabis by health care providers, such as the following participant, who worried about possible adverse effects on his other health conditions:

Cannabis, since it’s legalized, I have been thinking about it. The question I ask myself now, when I have pain, should I try cannabis? I’m afraid, and I talk about it with my doctors. They always say to me that I have only one kidney. When I talk about it with my doctor, they don’t agree so much because of my condition. (Male, 70–79, FG5)

#### A Therapy Rather than A Recreational Drug

All patients who used cannabis for their chronic pain insisted on the fundamental difference between their consumption and recreational use. Several patients mentioned their “youth times” when they used cannabis recreationally in totally different contexts than the present time. Many affirmed the importance of having a medical follow-up, and they valued the absence of psychotropic effects in medical cannabis use:

Regarding cannabis oil, the doctor decides the dose, and if you take it all in the first week, you can’t have it for the rest of the month. It’s very well managed and controlled. There is no hallucinatory effect in it. (Female, 60–69, FG3)

Patients generally preferred the effects of CBD over tetrahydrocannabinol (THC) because the latter was usually associated with psychotropic effects. Several of them emphasized that they took low dosages. The pharmaceutical form was also an essential element for these participants. Many of them explained that they did not want to smoke cannabis but preferred using oil or capsules, which have no recreational connotations:

Cannabis helps me. When I take it, there is practically no pain at all, it’s very surprising. And I do not take that much. I get it at [the cannabis clinic], there’s an office and I have it delivered. It’s CBD capsules; there is also a spray that can be used on the tongue. Anyway, I don’t want to smoke it, I’m not chasing the “high” effect. (Male, 50–59, FG4)

However, the lack of insurance coverage for cannabis by health care insurance sometimes jeopardized patients’ consideration of this treatment. For example, one participant reported that despite having medical authorization, she purchased cannabis from illegal sources because she could not afford the products sold in medical cannabis facilities:

I have a prescription, but I don’t purchase it from the companies that are supposed to be legal, because it’s too expensive. It’s way too expensive. [From illegal companies,] I can get, like, double the amount [for the same price]. But I know the product, and I know that’s what I have been prescribed, and I take the same dose. I just don’t order from the same place. (Female, 50–59, FG5)

### Pharmacists’ Perspectives

#### Monitoring Drug Interactions and Adverse Effects despite Scarce Scientific Data

Most pharmacists in the study pointed out the scarcity of evidence about the efficacy and safety of cannabis for pain treatment, which caused many uncertainties in their practice. Pharmacists were particularly concerned with the possible interactions of cannabis with other medications. For many of them, drug interactions were the primary cause of their reluctance and uncertainty, given the diversity of the available cannabis products:

Interactions are not well known with cannabinoids; we are beginning to learn about some interactions, but it’s still not well known. The administration route also changes the interactions: if it’s smoked, you’re going to have interactions with certain cytochromes that you won’t have if the product is taken orally or by cream or things like that. There are 1000 or more cannabis products; each company has different subtypes of cannabis with different types of THC, CBD, so it’s super complex to find your way around. The studies are mostly inconclusive. (Male, 14 years of practice, FG7)

Several pharmacists explained that they tried to prevent the most frequent adverse effects of cannabis products by targeting at-risk patients. However, many felt that they had not received sufficient information and training to adequately manage patients treated with cannabis:

There are certain contraindications. For psychiatric patients who have chronic pain, we obviously need to be more careful; a patient who has just had a heart attack, or who has heart failure, we’ll be more careful in recommending cannabis because it causes a lot of tachycardia, but apart from that, we’re in a bit of a limbo because there are so few studies, and we haven’t been trained in this area, so it’s hard to make up our minds. (Male, 4 years of practice, FG10)

Furthermore, some pharmacists found that it was hard to find their place given that they were not officially involved in the dispensing of most medical cannabis products sold in specialized facilities. They broadly referred to noteworthy time and resource obstacles when it comes to genuinely committing to extra activities. This could further complicate their task of preventing drug interactions and adverse effects. Indeed, they considered that they still had “a role to play,” but some of them underlined that their exact role was not clear, including for other health care providers:

Even in the medical community, there’s a little bit of confusion about who manages that. Do we need to refer patients to the SQDC [Société québécoise du cannabis; i.e., the provincial licensed recreational cannabis stores]? Is there another organization? Personally, I received a prescription for cannabis, and I had to tell the patient: “It’s not us who manage it,” and his nurse was like: “Oh, but I thought it was the pharmacist,” and the doctor didn’t really know either. So, sometimes, the patient is in the middle of all that and goes: “I don’t know what to do!” So even in the medical field, with legalization, there’s a bit of confusion as to how to provide this product, how to properly manage it. (Male, 5 years of practice, FG10)

The roles and activities of pharmacists in this regard were previously discussed in our broader qualitative study.^19^

#### Informing and Treating Patients Self-Medicating with Cannabis amid Its Growing Popularity

Many pharmacists noticed that since recreational cannabis was legalized in 2018, they have received more and more questions and queries from patients seeking information about medical cannabis. Some pharmacists mentioned a “fad phenomenon,” which they linked with the increased presence of cannabis in the mainstream media and on the internet. These participants deplored the misleading marketing strategies of cannabis companies that made it appear as a miracle cure. They reported that their patients were often exposed to all sorts of information that did not always reflect the scientific evidence. Therefore, many pharmacists considered that their role was to inform patients and prevent disproportionate expectations:

What I often try to explain to patients is that there’s not much evidence for cannabis in chronic pain. Yes, it works for some patients, but it’s not a panacea either. Often, people see videos on the internet that cannabis is really THE thing that works for chronic pain, but in reality, this is not always the case, and there are still significant side effects such as drowsiness but also mental health issues. (Female, 3 years of practice, FG8)

Several pharmacists believed that the recreational cannabis legalization increased both the demand and the supply of cannabis products. They reported that patients used a wide range of cannabis products to self-medicate their pain, which could make it more difficult for pharmacists to prevent unpredictable effects:

Often, the patient comes to the medical clinic, or to the community pharmacy, [and] they’ll say: “I tried this.” I’ve had a case of a cannabis beer that the person brought in to try for their wife [who had] chronic pain, but in the end, she just got buzzed—excuse the expression—she got buzzed, she got bad-tripped—you often see such effects with that. The nonpredictability of the effect is one of the constraints. Given that it has been decriminalized recently, we still lack feedback. (Male, 7 years of practice, FG9)

Nonetheless, many pharmacists explained that since recreational cannabis was legalized, it was easier for them to ask patients about their cannabis use. They felt less embarrassed about this topic that was sometimes included in routine medical questions. They also felt that it was easier to get an accurate answer from patients. Cannabis use was a less sensitive issue because it did not refer to illegal practices anymore. This facilitated their work of informing patients and preventing potential risks:

Since it’s become legal, we’ve become comfortable asking if they use cannabis, because before it was legal, if we asked the question, “Do you use cannabis?,” we weren’t sure if the answer was yes or a real no, you know. So now that it’s legal, I think people are becoming more comfortable with telling us the truth. That’s important for our pharmacological analysis as well. (Male, 10 years of practice, FG8)

Importantly, several pharmacists found that cannabis was useful for some of their patients. When cannabis successfully improved patients’ pain condition, several pharmacists insisted on the importance of officializing a treatment initiated through self-medication to have better control on the product’s composition:

In pain management, cannabis is often an additional tool. If the person responds well to their cannabis, it can be interesting to legalize that, to have a prescribing doctor for that, and more controlled products and doses that we increase gradually. (Female, 7 years of practice, FG11)

#### Financial Costs and Legal Constraints for Patients

Several pharmacists reported that their patients’ experiences with medical cannabis were overall ambivalent due to an imbalanced cost–efficacy ratio. Some pharmacists pointed out that the high financial cost was one important reason why their patients stopped cannabis treatments. One of them highlighted the socioeconomic inequities influencing the access to and outcomes of medical cannabis:

My only patients for whom it [cannabis] works well are those who have money [laughs], those who can afford the products that are useful for them. For those who can’t afford it, often I start by talking about the price, because sometimes it makes the consultation really last less time. (Male, 14 years of practice, FG7)

Another participant mentioned that specific legal constraints regulating medical cannabis, such as travel restrictions, could also dissuade patients from considering this treatment:

For this patient, not being able to travel with medical cannabis was an obstacle. I consider that it was like an adverse effect that had prevented the patient from starting the treatment. (Female, 2 years of practice, FG7)

## Discussion

This qualitative study advances knowledge about patients’ and pharmacists’ variable expectations and uncertainties as to the use of cannabis in chronic pain treatment in a context where both medical and recreational cannabis are legal. Considering the double perspective of both patients and pharmacists provided a strong added value to existing research in this field. Indeed, our work highlighted several convergences and divergences between patients’ and pharmacists’ views, practices, and concerns regarding cannabis. In this section, we discuss our original contributions to the literature and their implications for policy and practice.

Many patients in this study tended to compare cannabis with other pain medications to appraise its benefits and risks. Several patients reported that cannabis was a suitable solution to help them taper or cease more burdensome pain treatments such as opioids or antidepressants. Conversely, pharmacists were particularly concerned with the risks for interactions between cannabis and other medications. Pharmacists viewed cannabis as a complementary product to the patients’ previous pain medications, whereas patients considered it as a potential alternative to these medications. These diverse perspectives regarding the role of cannabis in patients’ treatment created contrasts between some patients’ positive views and pharmacists’ uncertainties about managing medical cannabis. As in our study, previous research highlighted that patients saw medical cannabis as a safer product compared to “conventional” pain medications^9,28^ and a potential alternative to opioids.^10^ A qualitative exploration of the co-use of cannabis and opioids showed that primary care physicians and nurses were concerned about the negative outcomes of cannabis on mental health, whereas patients were more concerned about addiction risks but valued the benefit of using both cannabis and opioids in terms of pain relief.^29^

The perspectives of pharmacists in our study underscored the urgent need for clinical and observational studies evaluating the efficacy and safety of cannabis, especially its interactions with other medications, to support appropriate benefit–risk appraisal. Such assessment is essential given that some recent pain guidelines tend to recommend cannabis as an adjunctive therapy only.^30^ According to pharmacists in this study, the legalization of recreational cannabis in Canada in October 2018 had a positive effect on their dialogue with patients, easing their access to information on patients’ practices with cannabis. However, without reliable data on the safety of cannabis and drug interactions, pharmacists’ ability to prevent cannabis-related harms is diminished. Importantly, because pharmacists are not involved in medical cannabis dispensing in Canada, it is crucial to provide them with the most up-to-date information on patients’ use so they can have the full picture and detect drug interactions and adverse effects. Pharmacists are first-choice professionals to be involved in benefit–risk assessment and pharmacovigilance to increase the safety of cannabis use for chronic pain.

Both patients and pharmacists in our study expressed concerns regarding the lack of scientific evidence and the relative recency of cannabis products in the treatment of pain. Our results gave a more nuanced picture than previous literature by highlighting not only patients’ positive views but also patients’ reluctance to use cannabis and their concerns about potential risks for their health. Pharmacists worried about patients’ self-medication practices in the context of an increasingly diverse supply of cannabis products and growing exposure to overly positive information. Pharmacists highlighted the challenge of providing patients with reliable scientific data when the current research evidence is scarce and not easily available. The few available quantitative data describing pharmacists’ opinions on medical cannabis in the United States and Canada also showed that most pharmacists felt unprepared and inadequately trained.^31,32^ Pharmacists expressed the need for training about local regulations on medical cannabis and the pharmacological properties of cannabis products.^31^ Another study showed that a cannabis course had a positive impact on pharmacy students’ confidence with patients using cannabis and on their vigilance in preventing associated risks.^33^ Our results, added to these previous studies, underscore the importance of developing access to evidence-based information about cannabis for both patients and pharmacists. Easily understandable resources for patients about the state of scientific knowledge on the benefits and harms of cannabis would help address patients’ fears and uncertainties, as well as some patients’ expectations for a miracle cure. Initial and continuous training resources tailored to pharmacists’ needs are also essential to supporting their daily practice with patients using cannabis for chronic pain.

Our study contributes to better understanding the practices and choices of patients with chronic pain regarding cannabis products. Patients in this study were more inclined to use CBD products rather than THC products, taken orally (oil, capsules) rather than smoked, and in lower dosages. They wanted to avoid psychotropic effects and did not wish to be confused with recreational users. These findings suggest that some cannabis products and usages connote recreational purposes and are still less socially acceptable despite legalization. Other research provides complementary quantitative data suggesting that patients’ preferences vary depending on the intensity of use, with heavy users of medical cannabis showing a greater preference for smoking or vaporizing and for higher THC content compared to light users, who prefer CBD and nonsmoking administration routes.^34^ Females and novice users are also more likely to report avoiding smoked cannabis and THC.^13^

Finally, the cost of medical cannabis and its specific dispensing system in specialized facilities were mentioned by both patients and pharmacists as potential barriers to access. Several participants highlighted the still specific status of medical cannabis, which is neither covered by insurance nor delivered in pharmacies, unlike medications. For patients, this could lead to illegal supply strategies, which raises safety issues due to the unregulated composition of illegal cannabis products.^35^ Pharmacists reported that the cost of medical cannabis dissuaded some of their patients from trying or continuing this treatment. Indeed, according to Statistics Canada, in September to December 2019, the average price of legal cannabis was CA$10.30 per gram, whereas the estimated average price of illegal cannabis was CA$5.73 per gram.^36^ This further highlights the urgent need to develop sufficient clinical evidence on the benefits and risks of cannabis to explore its coverage by public and private insurance for persons living with chronic pain who have explored all other treatment options.

Pharmacists in our study also reported some challenges with the dispensing system of medical cannabis in Canada, which does not involve their participation. These dispensing conditions could hinder pharmacists’ capacity to support patients as well as their access to information about patients’ cannabis treatments. It also caused misunderstandings with some prescribers who were not aware of the pharmacist’s limited role. In an Australian study, pharmacists were in favor of the dispensing of medical cannabis in community pharmacies to increase accessibility of standardized and legal cannabis products for patients.^37^ However, our study and previous research^19^ suggests that although dispensing medical cannabis in community pharmacies could benefit both patients and health care providers, it will be important to support pharmacists in this mission by providing them with sufficient material resources and training.

### Study Limitations

As in any study using focus groups, the specific dynamics of group discussions may have influenced data collection. It is essential to acknowledge that focus groups are known to encourage consensus,^38^ so we cannot rule out that some participants thus did not express divergent opinions. However, we were able to collect diverse perspectives on cannabis (e.g., both positive and negative views) across the different focus groups, which provides nuance in the narratives and fosters the dependability of our data. Moreover, it is noteworthy to acknowledge the distinctive role of the researcher in qualitative inquiries as an integral component of the research project. In fact, in alignment with reflexivity,^39^ our position and perspective as researchers influenced every step of the research process (relation between interviewers and interviewees, transcription of interviews, interpretation). Conducting a large number of focus groups also helped us mitigate this potential issue. Another potential limitation could be related to participants’ sociodemographic characteristics. The sample is relatively homogenous in terms of age (older for patients and younger for pharmacists) and ethnicity (all but five participants identified as white). However, recruitment included participants from urban, rural, and remote areas, as well as both women and men, which increases the ability of our results to adequately represent diverse experiences. The perspectives of participants from racial and ethnic minorities would certainly enrich our findings.

## Conclusion

This qualitative study highlighted the hopes and uncertainties of patients and pharmacists regarding cannabis use for chronic pain. It is essential to develop evidence on the benefits and risks of cannabis for chronic pain treatment, including drug interactions and adverse effects. Training interventions for pharmacists should be implemented to support them in providing their patients with accurate information regarding cannabis use in chronic pain treatment. Knowledge translation tools and information campaigns for the general public could also help address patients’ expectations and concerns and improve their experience through fostering safe medical cannabis use.
